# Interaction between high-temperature magmatic fluids and limestone explains ‘Bastnäs-type’ REE deposits in central Sweden

**DOI:** 10.1038/s41598-019-49321-8

**Published:** 2019-10-23

**Authors:** Fredrik Sahlström, Erik Jonsson, Karin Högdahl, Valentin R. Troll, Chris Harris, Ester M. Jolis, Franz Weis

**Affiliations:** 1 Department of Earth Sciences, Uppsala University, SE-75236 Uppsala, Sweden; 2 Department of Mineral Resources, Geological Survey of Sweden, SE-75128 Uppsala, Sweden; 3 Department of Geological Sciences, University of Cape Town, 7701 Rondebosch, Republic of South Africa; 4 Institut für Mineralogie, Westfälische Wilhelms-Universität Münster, 48149 Münster, Germany

**Keywords:** Geochemistry, Geology, Mineralogy, Petrology, Volcanology

## Abstract

The presently increasing demand for rare earth elements (REE), particularly in high-tech and “green energy” applications, has led to global interest in the distribution, origins and formation conditions of REE deposits. The World’s first hard-rock REE sources, the polymetallic deposits of Bastnäsfältet in Bergslagen, central Sweden, were also the place of the original discovery of several REE and many of their host minerals. Similar deposits with high concentrations of REE occur along a > 100 km corridor in the region and they share a number of geological and mineralogical features; all comprising Palaeoproterozoic, skarn-hosted magnetite-REE mineralisation of ambiguous origin. Here we report oxygen isotope data for magnetite and quartz, and oxygen and carbon isotope data for carbonates from ten of these classic deposits, to model and assess their mode of origin. Combined with existing geological observations, the isotope results support an origin in a c. 1.9 Ga shallow-marine back-arc, sub-seafloor setting, where felsic magmatic-sourced, high-temperature fluids reacted with pre-existing limestone interlayers, leading to localised skarn formation and magnetite-REE-mineral precipitation. These findings help us to better understand the geological processes that have produced economic REE mineralisation and may assist future exploration for these critical commodities.

## Introduction

**Figure 1:**
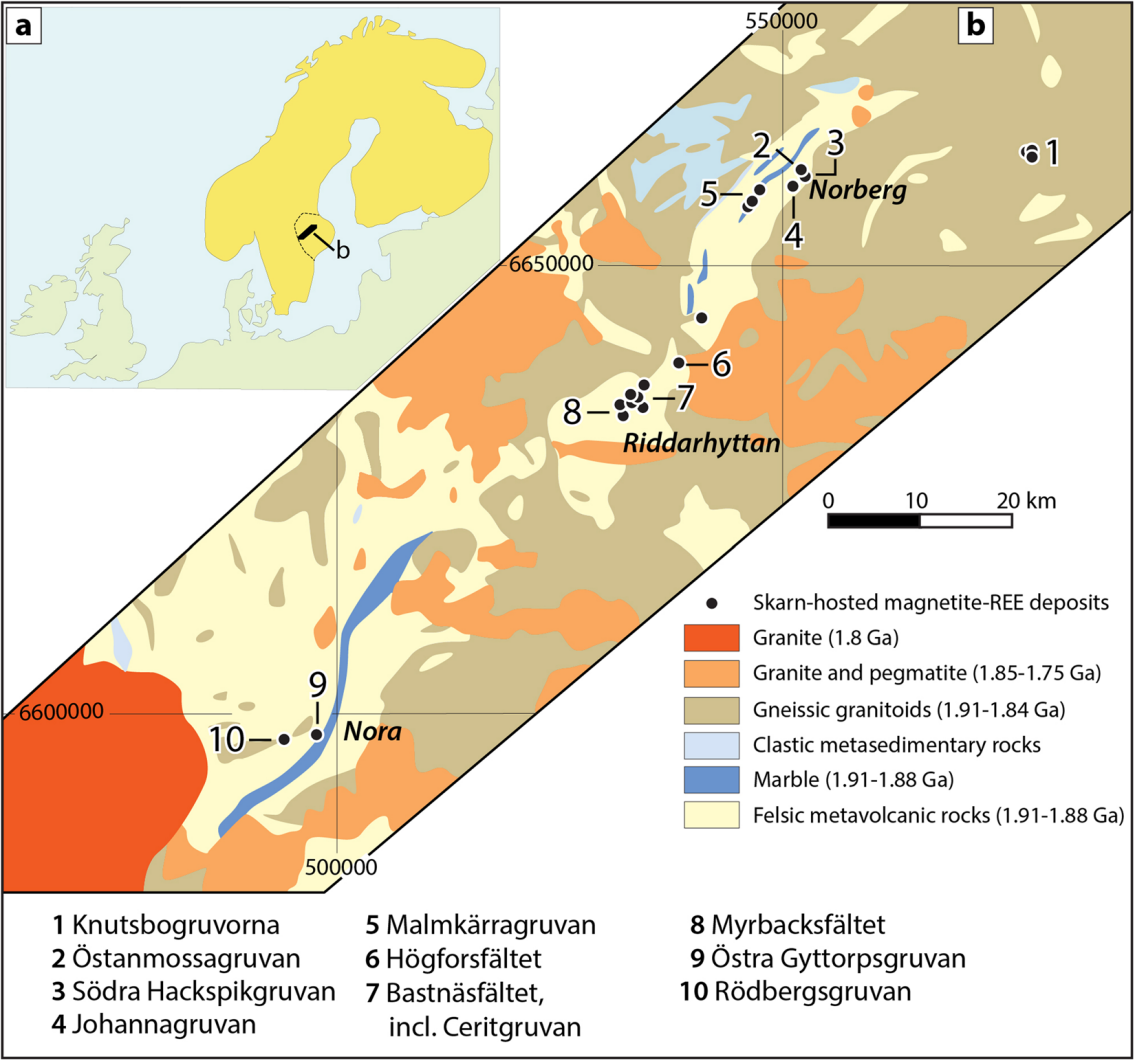
Maps and sampling locations. (**a**) Overview map of the Fennoscandian Shield and part of the Caledonian orogen (yellow), with the Bergslagen ore province (stippled line) and the REE-line (black) indicated. (**b**) Geological map of the REE-line, Bergslagen, central Sweden. The ten Bastnäs-type deposits sampled in this study are marked. The towns of Nora, Riddarhyttan and Norberg are included as landmarks. Grid is Swedish national grid RT90.

The rare earth elements (REE) are critical components in a vast range of modern high-tech products and technologies. These include many types of industrial, research and consumer electronics, as well as increasingly abundant “green energy” applications such as wind power and electric vehicles^1–5^. Economic projections suggest an even greater demand for REE in the immediate future, especially since numerous countries aim at phasing out fossil fuel-based transportation and energy production^6,7^. This demand, combined with the present lead of China as the main supplier of REE, has led to a renewed global interest in REE deposits of various types and geological origins. In Europe, one of the regions with the largest exploration potential for critical metals such as the REE is the Fennoscandian Shield (Fig. 1), where a multitude of REE-bearing mineralisations occur^8^. Of these, one of the most iconic, yet puzzling ore types is the REE-enriched magnetite skarns in the Palaeoproterozoic Bergslagen ore province in south central Sweden (Fig. 1). While not being mined at present, their presence and high grades have warranted recent and on-going exploration for REE in the area.

The mines of Bastnäsfältet (*the Bastnäs field*) in Bergslagen (Fig. 1), and in particular Ceritgruvan (*the cerite mine*), was from the mid-19^th^ century where the first hard-rock underground mining was performed to extract REE^9^. The main REE minerals in the ore are cerite-(Ce) [(Ce,LREE,Ca)_9_(Mg,Fe)[SiO_4_]_6_[SiO_3_OH](OH)_3_] and ferriallanite-(Ce) [Ca(Ce,LREE)Fe^3+^AlFe^2+^[SiO_4_][Si_2_O_7_]O(OH)]. Moreover, Bastnäsfältet was also the location of the original discovery of cerium, lanthanum, and “didymium” (a mixture from which praseodymium and neodymium were later isolated)^10–12^, as well as numerous minerals, including the already mentioned cerite and the eponymous bastnäsite-(Ce) [(Ce,LREE)(CO_3_)F]^11,13^. After the initial discovery at Bastnäsfältet by Hisinger^14^, bastnäsite was to become one of the World’s major sources of REE^4^. As additional mineralisations were recognised to have a similar character as those at Bastnäsfältet, the term “Bastnäs-type deposit” was coined^15,16^. The Bastnäs-type deposits are characterised by skarn-hosted magnetite-REE mineralisation, with or without additional polymetallic assemblages^11,15,17^. These deposits occur as clusters along the so-called “REE-line”^18^; a more than 100 km long, narrow belt in the western Bergslagen region of variably Na, K and/or Mg altered, c. 1.90–1.88 G.y. old felsic metavolcanic rocks with intercalated marble layers (Fig. 1). This volcano-sedimentary sequence formed during magmatic activity in what has been interpreted as a shallow-marine, but principally continental back-arc setting^19^. Both the magnetite-REE ore bodies and the host rocks have been affected by later, polyphase deformation and greenschist to amphibolite facies metamorphism at c. 1.85–1.80 Ga during the Svecokarelian orogeny^20–23^. In the Bergslagen ore province, where base metal mineralisations and iron oxide ores predominate^23–27^, the REE-line represents both a geological anomaly and a zone of significant concentration of in general light REE, but also yttrium, and the heavy REE^16^. The REE minerals in these deposits are dominantly silicates [allanite- and dollaseite-group minerals, cerite-(Ce), fluorbritholite-(Ce), törnebohmite-(Ce)], and the carbonate bastnäsite-(Ce), besides numerous low-volume accessory phases^16,17^. The skarns hosting the magnetite-REE mineralisations are calc-silicate aggregates, typically amphibole-dominated, featuring actinolitic to tremolitic, as well as anthophyllitic compositions^28–30^. Magnetite was always the main ore mineral mined in these deposits, besides the REE mineralisations, but copper as well as cobalt ores have also been locally exploited^29,30^.

The genesis of the Bastnäs-type REE deposits has been critically debated with respect to their formation process, and their temporal relationships to host rocks and to other mineralisation types^11,15–18,28,30,31^. Their genesis, and that of the associated skarn iron ores and extensive host rock alteration, was originally interpreted to be related to large-scale, so-called “magnesia metasomatic” processes, generated by abundant granitoid intrusions during the waning stage of regional (Svecokarelian) metamorphism^15,27^. Later studies have rendered this theory obsolete^17,18,31,32^, and they mostly revolve around an essentially syn-volcanic hydrothermal formation scenario at c. 1.90–1.88 Ga, or somewhat later. Here we employ mineral oxygen and carbon isotopes to unravel the fundamental process involved in the formation of the Bastnäs-type REE deposits, and to provide robust constraints on the temperature and source(s) of ore-forming fluids.

## Results and Interpretation

**Figure 2:**
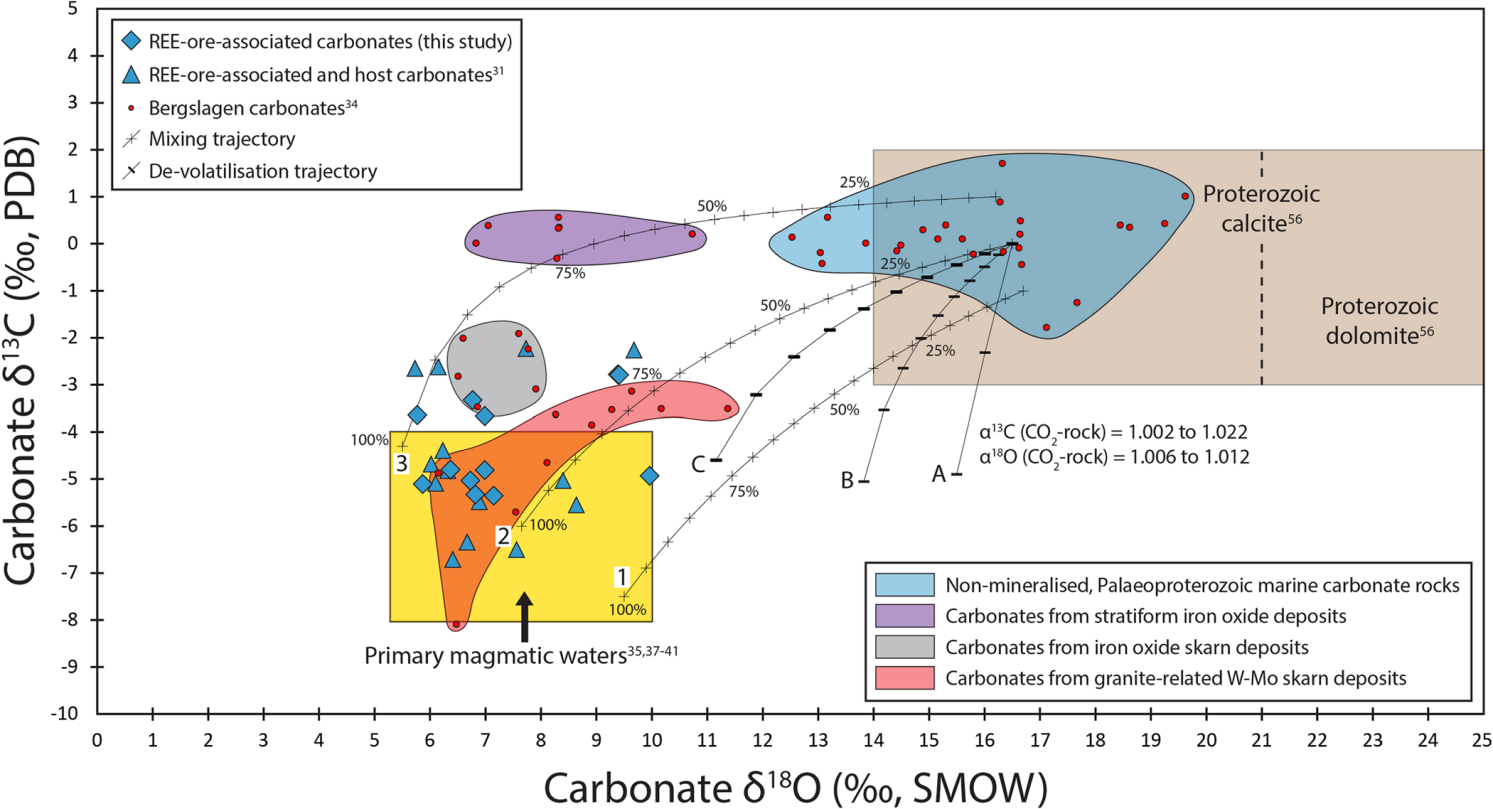
Distribution of δ^18^O and δ^13^C values and numerical models for carbonates from Bergslagen. The analysed carbonates associated with magnetite-REE mineralisation in Bastnäs-type deposits (blue diamonds; this study) and complementary data (blue triangles) from Holtstam *et al*.^31^ are shown. Also shown are literature data for carbonates from the Bergslagen province (red dots)^34^, encompassing non-mineralised, Palaeoproterozoic marine carbonate rocks (blue field), carbonates from stratiform iron oxide deposits (purple field), carbonates from iron oxide skarn deposits (grey field), and carbonates from granite-related tungsten-molybdenum (W-Mo) skarn deposits (red field). Reference fields for Proterozoic marine calcite and dolomite (brown field)^56^ and primary magmatic waters (yellow field)^35,37–41^ are also included. Trajectories A-C represent Rayleigh-type de-volatilisation of local, non-mineralised marine carbonate rocks of average composition, using the indicated fractionation factors^35^. Lines 1–3 are binary mixing trajectories at 5% intervals between local marine carbonate rocks and a typical magmatic aqueous fluid composition. The calculated magmatic fluid-carbonate mixing trajectories envelop essentially all of the analysed carbonates from the Bastnäs-type deposits. See text for detailed explanation.

Representative, variably mineralised rock samples were both collected in the field and sourced from the collections of the Geological Survey of Sweden in Uppsala and the Swedish Museum of Natural History in Stockholm. The sample suite encompasses ten different Bastnäs-type deposits from within the REE-line, Bergslagen (Fig. 1), and comprises massive magnetite ore (from Bastnäsfältet, Myrbacksfältet, Östra Gyttorpsgruvan and Rödbergsgruvan), skarn and skarn ore with disseminated-type magnetite (from Knutsbogruvorna, Östanmossagruvan, Södra Hackspikgruvan, Johannagruvan, Malmkärragruvan and Bastnäsfältet) and magnetite-skarn-bearing banded iron formation (BIF) ore (from Högforsfältet; Table 1). Magnetite concentrates (n = 25) were separated from all the ore types and have δ^18^O values ranging from −2.3 to +2.6‰, whereas co-existing quartz (n = 3) separated from the BIF-hosted magnetite-skarn assemblages have δ^18^O values between +7.2 and +8.3‰ (Table 1). Carbonate minerals (n = 12) were separated from a suite of the variably magnetite- and REE-bearing skarn assemblages. The carbonate separates comprise both calcite [CaCO_3_] and dolomite [CaMg(CO_3_)_2_], as well as mixtures between the two, and have δ^18^O values from +5.8 to +10.0‰ and δ^13^C values from −5.4 to −2.8‰ (Table 1, Fig. 2).

**Table 1:** Mineralogical characterisation and oxygen and carbon isotope data for samples from Bastnäs-type deposits, REE-line, Bergslagen.

Sample ID	Deposit	Type	Mineral Association^a^	%_Dol_^b^	δ^18^O_Mgt_	δ^18^O_Qtz_	δ^18^O_Cb_	δ^13^C_Cb_	Qtz-Mgt Temp.
NRM-20020125	Knutsbogruvorna	skarn-magnetite	Qtz, Mgt, Hem, Am, Py, Dla-(Ce), Aln-(Ce)		−0.7	7.5			505 ± 22 °C
NRM-20020124	Knutsbogruvorna	skarn-magnetite	Qtz, Mgt, Hem, Am, Dla-(Ce)		−1.9				
EJ-OM90-13-1	Östanmossagruvan	skarn-magnetite	Cb, Am, Fl, Mgt, Dla-(Ce), Flb-(Y), Bas-(Ce), Par-(Ce), Mon-(Ce), Urn, Scl	0	−1.2		7.2	−5.4	
SGU-M4528	Östanmossagruvan	skarn-magnetite	Cb, Am, Fl, Flb-(Y), Dla-(Ce), Mgt, Bas-(Ce), Par-(Ce), Hu	100	−1.4		5.8	−3.6	
EJ-OM90-13-2	Östanmossagruvan	skarn	Cb, Am, Dla-(Ce), Bas-(Ce), Flb-(Y), Hu, Urn	10			6.4	−4.8	
SGU-M441	Östanmossagruvan	skarn	Cb, Am, Flb-(Ce), Dla-(Ce), Fl, Bas-(Ce), Par-(Ce), Hu	20			6.8	−5.3	
SGU-M4529	Östanmossagruvan	skarn	Cb, Am, Fl, Dla-(Ce), Flb-(Y), Bas-(Ce), Par-(Ce), Gad-(Y), Hu	85			5.9	−5.1	
EJ-OM14-1	Östanmossagruvan	skarn-magnetite	Cb, Dla-(Ce), Mgt, Am	9	2.6		9.4	−2.8	
EJ-OM14-2	Östanmossagruvan	skarn-magnetite	Cb, Dla-(Ce), Mgt, Am	10	−0.9		7.0	−4.8	
EJ-OM14-3	Östanmossagruvan	skarn-magnetite	Cb, Dla-(Ce), Mgt, Am	9	−1.5		6.7	−5.0	
SGU-M3563	Södra Hackspikgruvan	skarn-magnetite	Fl, Am, Cb, Mgt, Dis-(Ce), Cer-(Ce), Bas-(La/Ce), Flb-(Ce), Py, Hu, Scl		0.5				
KH-Joha-1	Johannagruvan	skarn-magnetite	Am, Dla-(Ce), Mgt, Qtz, Bas-(Ce), Cp, Py, Sp, Gad-(Nd), Par-(Ce)		−0.5				
KH-Joha-2	Johannagruvan	skarn-magnetite	Am, Nrb, Dla-(Ce), Mgt, Qtz, Cp, Py, Bas-(Ce), Gad-(Nd), Par-(Ce), Mlb		0.5				
SGU-M4068-B	Malmkärragruvan	skarn-magnetite	Cb, Am, Fl, Väs-(Ce), Flb-(Ce), Par-(Ce), Bas-(Ce), Urn, Ulf-(Ce)	80	−0.6		6.8	−3.3	
SGU-M4048	Malmkärragruvan	skarn-magnetite	Cb, Am, Fl, Väs-(Ce), Flb-(Ce), Par-(Ce), Bas-(Ce), Urn	70	0.2		7.0	−3.7	
EJ-MMK14-1	Malmkärragruvan	skarn-magnetite	Cb, Mgt, Am	11	1.6		9.4	−2.8	
EJ-MMK14-2	Malmkärragruvan	skarn	Cb, Mgt, Am, Py	3			10.0	−4.9	
KH-Högf-1	Högforsfältet	BIF	Mgt, Qtz, Hem, Am, Cer-(Ce), Aln-(Ce), Mnz-(Ce)		−1.7	8.3			424 ± 17 °C
KH-Högf-2	Högforsfältet	BIF	Mgt, Qtz, Hem, Am, Cer-(Ce), Aln-(Ce), Bas-(Ce), Gad-(Ce)		−0.4	7.2			544 ± 25 °C
EJ-Bast-1	Bastnäsfältet	skarn-magnetite	Am, Mgt, Fln-(Ce), Py, Sp, Cp, Mbd		−0.6				
SGU-M6777	Bastnäsfältet	massive magnetite	Mgt, Am, Fln-(Ce), Py		−1.2				
SGU-M309	Bastnäsfältet	skarn-magnetite	Am, Mgt, Fln-(Ce), Py, Sp, Cp, Mnz-(Ce), Mbd		0.0				
EJ-Myrb-1	Myrbacksfältet	massive magnetite	Mgt, Py, Cp, Am, Qtz,		0.7				
EJ-Myrb-2	Myrbacksfältet	massive magnetite	Mgt, Py, Cp, Am, Qtz, Aln-(Ce)		1.1				
EJ-Gytto-1	Östra Gyttorpsgruvan	massive magnetite	Mgt, Bt, Am, Aln-(Ce), Gad-(Y), Hng-(Y), Bas-(Ce), Par-(Ce), Urn, Zrn		−1.8				
EJ-Gytto-2	Östra Gyttorpsgruvan	massive magnetite	Mgt, Bt, Am, Aln-(Ce), Gad-(Y), Hng-(Y), Bas-(Ce), Par-(Ce)		−2.3				
EJ-Gytto-3	Östra Gyttorpsgruvan	massive magnetite	Mgt, Bt, Am, Aln-(Ce), Bas-(Ce), Par-(Ce), Urn		−2.1				
NRM-880071	Rödbergsgruvan	massive magnetite	Mgt, Am, Aln-(Ce), Bas-(Ce), Cer-(Ce), Mlb		1.1				
NRM-19984100	Rödbergsgruvan	massive magnetite	Mgt, Am, Aln-(Ce), Cp, Väs-(Ce), Bas-(Ce), Cer-(Ce)		0.6				

^a^Mineral abbreviations: Aln – allanite; Am – amphibole; Bas – bastnäsite; Bt – biotite; Cb – carbonate; Cer – cerite; Cp- chalcopyrite; Dis – dissakisite; Dla – dollaseite; Dol – dolomite;

Fl – fluorite; Flb – fluorbritholite; Fln – ferriallanite; Gad – gadolinite; Hem – hematite; Hng – hingganite; Hu – humite group minerals; Mbd – molybdenite; Mgt – magnetite; Mnz – monazite;

Nrb – norbergite; Par – parisite; Py – pyrite; Qtz – quartz; Scl – scheelite; Sp – sphalerite; Ulf – ulfanderssonite; Urn – uraninite; Väs – västmanlandite; Zrn – zircon.

^b^The proportion of dolomite relative to calcite in carbonate separates, based on XRD data.

### Thermometry

Thermometric calculations for magnetite-quartz oxygen isotope pairs (n = 3), using the fractionation factor of Zheng & Simon^33^, yielded a temperature range of 425 to 545 ± 25 °C (Table 1). Although only a small dataset, these temperatures are in agreement with published fluid inclusion data for bastnäsite-(Ce) from the REE-line. The fluid inclusions are moderately to highly saline (6–29 wt.% CaCl_2_) and show (uncorrected) homogenisation temperatures of 370 to 460 °C^31^. Considering that significant lithostatic pressures are likely to have prevailed at the depths of skarn formation (c.f.^19^), and that, in general, bastnäsite-(Ce) is paragenetically late relative to magnetite, the fluid inclusion homogenisation temperatures should represent lower-end estimates of the range of mineralisation temperatures. Thus, the combined oxygen isotope and fluid inclusion evidence suggest that primary magnetite-REE mineralisation in Bastnäs-type deposits formed, at least initially, from fluids with temperatures of ≥450 °C.

### Oxygen and carbon isotope modelling for carbonates

The carbonates from Bastnäs-type deposits have δ^18^O and δ^13^C values that are significantly lower than those of non-mineralised, Palaeoproterozoic marine carbonate rocks in the Bergslagen province (δ^18^O = +12.5 to +19.6‰, δ^13^C = −1.8 to +1.7‰^34^; Fig. 2). However, they plot partly within the fields for previously analysed carbonates from iron oxide skarns and carbonates from granite-related (metasomatic) tungsten-molybdenum skarns^34^ (Fig. 2). To test for possible process scenarios behind the observed shifts in carbonate isotopic compositions for the Bastnäs-type deposits, we calculated two different numerical models. Trajectories A–C in Fig. 2 represent Rayleigh-type de-volatilisation of non-mineralised, Palaeoproterozoic marine carbonate rocks of average composition. Rayleigh de-volatilisation is defined as (shown for δ^18^O below):

1 $${{\rm{\delta }}}^{18}{{\rm{O}}}_{{\rm{f}}}-{{\rm{\delta }}}^{18}{{\rm{O}}}_{{\rm{i}}}=1000\ast ({{\rm{F}}}^{{\rm{\alpha }}-1}-1)$$

where F is the molar fraction of oxygen that remains in the rock after de-volatilisation; α is the fractionation factor; and δ^18^O_i_ and δ^18^O_f_ are the initial and final oxygen isotope compositions of the rock, respectively^35^. This model is based on the so-called “calc-silicate limit“, which assumes that all carbon is released as CO_2_ while c. 60% of the oxygen remains in the rock if the reaction goes to completion^35^. Unlike what has locally been observed in other marble-skarn environments in Bergslagen (e.g.^36^), the analysed carbonates from the Bastnäs-type deposits do not plot on the steep or near-vertical trends of pure thermal decarbonation (A-C in Fig. 2). These results suggest that such reactions alone cannot realistically have produced the observed isotopic distribution. Lines 1–3 in Fig. 2, in turn, represent binary mixing trajectories between the same non-mineralised, Palaeoproterozoic marine carbonates and a typical magmatic aqueous fluid composition (δ^18^O = +5.3 to +10.0‰, δ^13^C = −8.0 to −4.0‰)^35,37–41^, using different end-member compositions. Mixing is defined as:

2 $${{\rm{R}}}_{{\rm{M}}}={{\rm{R}}}_{{\rm{A}}}{{\rm{X}}}_{{\rm{A}}}{\rm{f}}+{{\rm{R}}}_{{\rm{B}}}{{\rm{X}}}_{{\rm{B}}}\ast (1-{\rm{f}})/({{\rm{X}}}_{{\rm{A}}}{\rm{f}}+{{\rm{X}}}_{{\rm{B}}}(1-{\rm{f}}))$$

where R_M_ is the isotope ratio of element X in a mixture of compositions A and B; X_A_ and X_B_ are the concentration of X in A and B, respectively; and f is the weight fraction of A defined as f = A/(A + B)^41^. The calculated magmatic fluid-carbonate mixing trajectories envelop essentially all of the analysed carbonates from the Bastnäs-type deposits (Fig. 2). Based on these results, we interpret that progressive CO_2_ release during interaction between a magmatic-derived, aqueous hydrothermal fluid and local, marine carbonate horizons caused the observed shifts to lower δ^18^O and δ^13^C values in the primary (protolith) carbonates, as well as re-precipitation of secondary (hydrothermal) carbonates of similar compositions (cf.^35,42–44^). Such processes have, notably, been recognised at magnetite skarn deposits elsewhere (e.g. Turgai belt, Kazakhstan^45^) and at several magma-carbonate contacts (e.g. Vesuvius volcanic system, Italy^46^ and Merapi volcano, Indonesia^47^).

### Oxygen isotope fluid modeling

**Figure 3:**
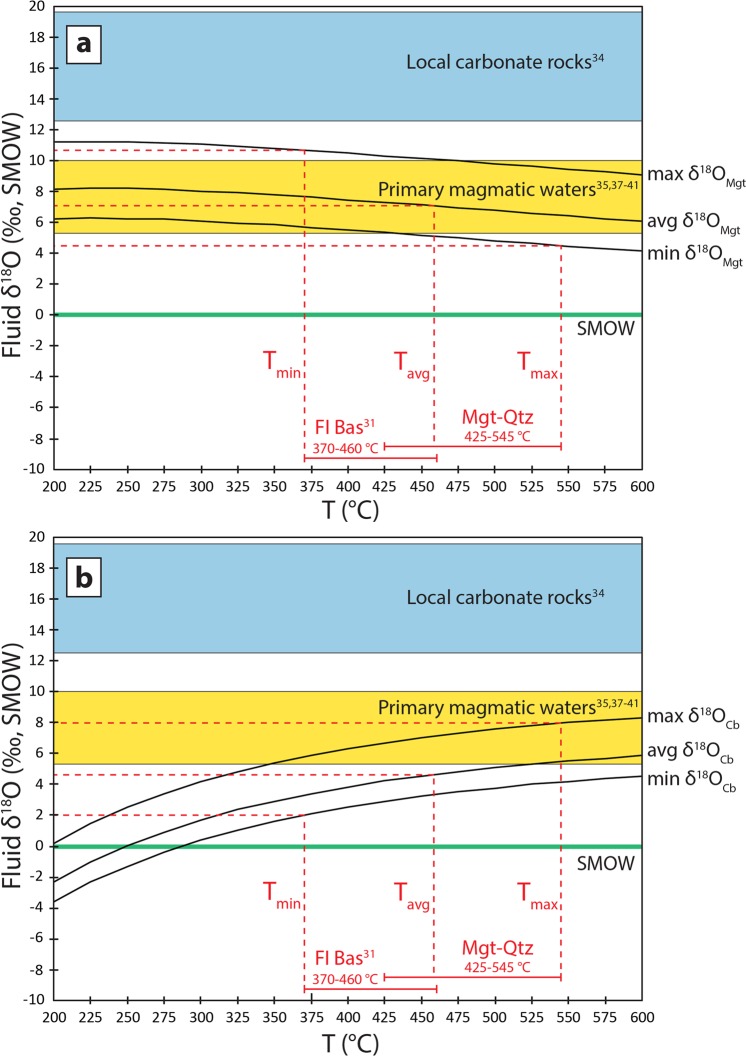
Oxygen isotope compositions of aqueous fluids in equilibrium with magnetite and carbonates from Bastnäs-type deposits at temperatures of 200 to 600 °C. (**a**) Equilibrium fluid compositions calculated based on the average, minimum and maximum δ^18^O values measured in magnetite, respectively. The red stippled lines indicate fluid compositions for specific temperatures, within the range given by magnetite-quartz oxygen isotope pairs and published fluid inclusion data for bastnäsite-(Ce)^31^. Reference fields include primary magmatic waters^35,37–41^, local carbonate rocks^34^ and the SMOW line. (**b**) Similarly modelled oxygen isotope compositions of equilibrium fluids for carbonates. The combined magnetite-carbonate data imply an initially high-temperature, magmatic-dominated hydrothermal system that evolved through decreasing temperature and an increasing input from non-magmatic, low-δ^18^O fluid sources. See text for detailed explanation. Abbreviations: Bas – bastnäsite; Cb – carbonate; FI – fluid inclusions; Mgt – magnetite; Qtz – Quartz; SMOW – Standard Mean Ocean Water.

Oxygen isotope compositions of hydrothermal aqueous fluids in equilibrium with magnetite from Bastnäs-type deposits were modelled for a temperature range of 200 to 600 °C (Fig. 3A), using the average (−0.4‰), minimum (−2.3‰) and maximum (+2.6‰) δ^18^O values measured in magnetite, respectively, and established fractionation factors^33^. For the proposed temperatures of mineralisation (c. 370 to 545 °C), fluids in equilibrium with magnetite of average composition have δ^18^O values ranging from +6.4 to +7.6‰ (Fig. 3A). These results are consistent with equilibration between magnetite and a magmatic-derived fluid (δ^18^O = +5.3 to +10.0‰)^35,37–41^. If the upper end of the temperature range is used, fluids in equilibrium with magnetite with the lowest δ^18^O value are slightly lower than primary magmatic waters (δ^18^O = +4.5‰; Fig. 3A). This trend may reflect mixing between original magmatic fluids and surface water with lower δ^18^O value. For the lower end of the temperature range, fluids in equilibrium with magnetite with the highest δ^18^O value are, in turn, higher than typical magmatic waters (δ^18^O = +10.7‰; Fig. 3A). Higher-than-magmatic fluid δ^18^O values are explained by interaction with pre-existing carbonate rocks (Fig. 3A).

The oxygen isotope compositions of hydrothermal fluids in equilibrium with carbonates from these deposits were also calculated for temperatures of 200 to 600 °C (Fig. 3B), based on the fractionation factors of Zheng^48^ and Sheppard & Schwarz^49^. Equilibrium fluid δ^18^O values in the range of primary magmatic waters, from +5.8 to +8.0‰, are only produced for the highest δ^18^O value (+10.0‰) of the analysed carbonates (Fig. 3B). The average (+7.4‰) and lowest (+5.8‰) carbonate δ^18^O values give significantly lower equilibrium fluid δ^18^O values. With decreasing temperature, they range from the lowermost limit of magmatic fluids down to +2.0‰ (Fig. 3B). Unlike the physically and chemically refractory magnetite, which is much more likely to retain its original chemical and isotopic composition (e.g.^50,51^), the reactive carbonates are more easily affected by both high-temperature processes that could lead to, e.g., de-volatilisation, and low-temperature fluid overprinting, either during primary skarn and ore formation or during subsequent phases of regional metamorphism and granitoid intrusion. Such retrograde isotopic modification may be more marked in some carbonates than in others, explaining the variation in carbonate equilibrium fluid δ^18^O values (Fig. 3B) relative to the more limited, essentially magmatic range for magnetite fluids (Fig. 3A).

Overall, the gradual transition from higher to lower equilibrium fluid δ^18^O values observed in the combined magnetite-carbonate datasets suggests that they record the progressive evolution of a hydrothermal system that commenced with high-temperature, magmatic-dominated fluids, via decreasing temperatures and gradual input (dilution) from external, non-magmatic water sources.

## Conclusions

**Figure 4:**
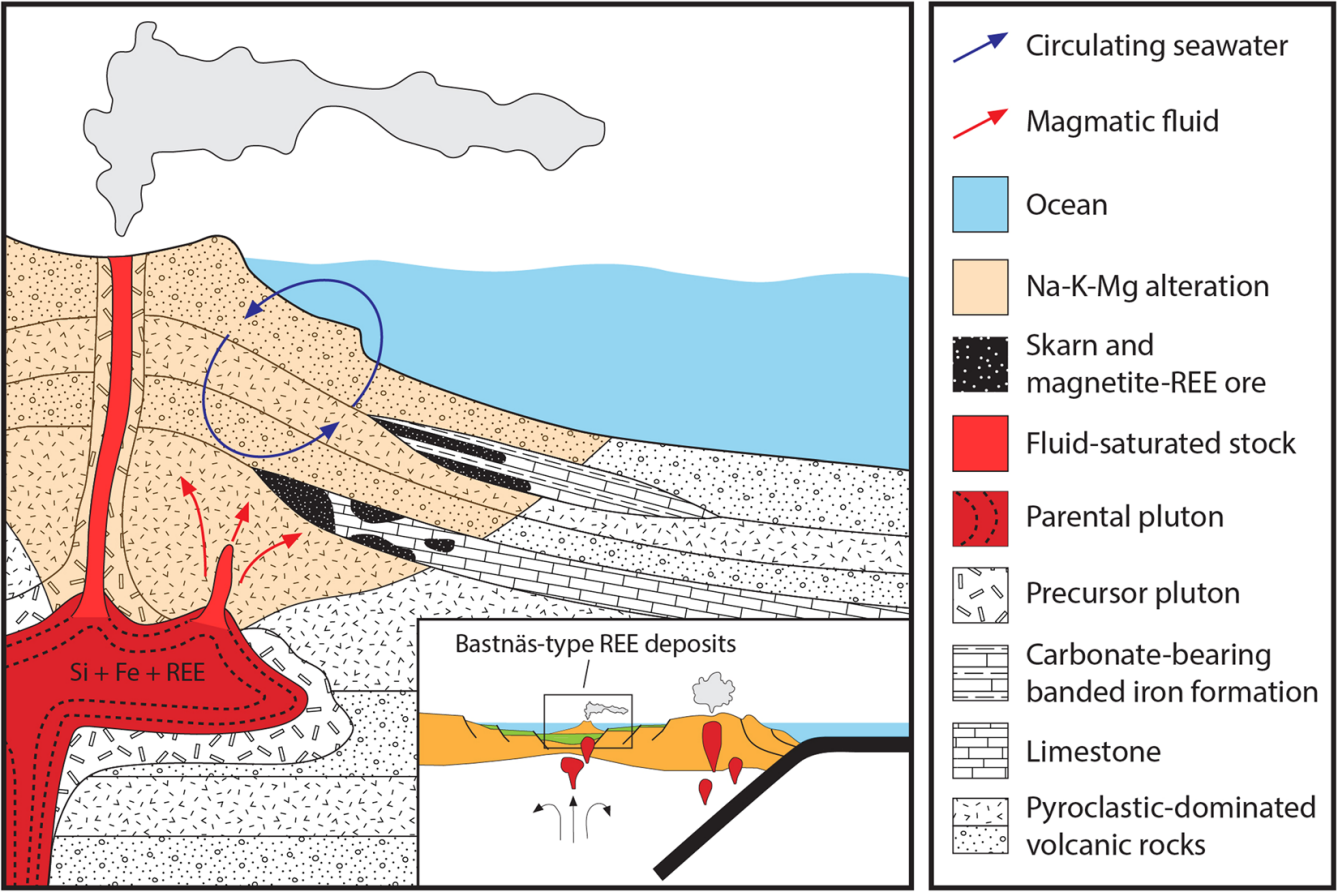
Cartoon model illustrating a likely scenario during the formation of the Bastnäs-type REE deposits in Bergslagen. These deposits are interpreted to have formed in a c. 1.9 Ga shallow-marine back-arc, sub-seafloor setting associated with extensive felsic volcanism and plutonism. High-temperature hydrothermal fluids, enriched in silica, iron and REE among other components, exsolved from a sub-volcanic magma and reacted with nearby interlayers of limestone and carbonate-bearing BIF. This led to skarn formation and magnetite-REE-precipitation within the carbonate units, while extensive hydrothermal alteration affected the surrounding volcanic host rocks. Over the life of the hydrothermal system there was progressive involvement of surface waters. See text for detailed explanation.

The new oxygen and carbon isotope data together with the generated numerical models demonstrate that the magnetite-REE-mineralised skarn assemblages from Bergslagen formed from high-temperature hydrothermal fluids of a predominantly magmatic origin. These fluids reacted with local, Palaeoproterozoic marine carbonate rocks and, over time, the hydrothermal system cooled and experienced influx of isotopically distinct (low-δ^18^O) water sources, such as seawater. Combined with available geological, geochronological and textural observations^15–19,23,28–32^, the new results are most easily reconciled with a scenario involving sub-seafloor, felsic magmatic activity at c. 1.90–1.88 Ga, within a shallow-marine back-arc setting (Fig. 4). In this setting, magmatic-sourced, metal- and silica-rich hydrothermal fluids were introduced to, and reacted with limestone interlayers in an otherwise pyroclastic-dominated volcano-sedimentary succession, leading to skarn formation and magnetite-REE-mineralisation.

Our study provides new evidence for a magmatic origin of the World’s original hard-rock source of REE, the Bastnäs-type REE deposits of central Sweden. These findings help us to better constrain the geological processes associated with formation of economic REE mineralisation, and will thus assist exploration for these critical commodities in the future. Specifically, the Bastnäs-type deposits represent a large-scale (>100 km) feature of high-grade REE concentration in the Bergslagen province (Fig. 1), but are currently unknown in the form of direct analogues from other locations globally. We propose that geological terranes elsewhere that constitute shallow-marine, sub-seafloor settings within continental back-arcs may be prospective for Bastnäs-type REE mineralisation. In such settings, evidence of extensive felsic magmatism combined with the presence of nearby carbonate horizons constitute key first-order exploration criteria (Fig. 4). Determining the cause of original REE enrichment in the magmatic systems that produced Bastnäs-type deposits, and whether or not it is unique to this tectonic setting, remains an important avenue for future research.

## Methods

### Mineralogical characterisation

Representative rock samples were cut and prepared as polished thin and thick sections, and subjected to transmitted and reflected polarised light microscopy and reconnaissance scanning electron microscopy with an energy dispersive scanning system (SEM-EDS). In selected cases, subsequent field emission electron microprobe analyses (FE-EPMA) were performed on a JEOL JXA-8530F Hyperprobe at the Department of Earth Sciences, Uppsala University.

To determine the composition of carbonate separates prior to stable isotope measurements, samples were subjected to powder X-ray diffraction (XRD) analysis at the Swedish Museum of Natural History, Stockholm. The samples were first crushed to a fine powder and placed in a silicon holder. They were then analysed using a PANalytical X’pert PRO automated diffractometer, utilising an acceleration voltage of 45 kV and a beam current of 40 mA. The 2θ angles were measured in the interval 5–70° for 11 minutes, and mineral identification was done off-line using the Highscore Plus software.

### Stable isotopes

Stable isotope data were produced at the University of Cape Town, South Africa. Magnetite and quartz separates were analysed following the laser fluorination technique described in Harris & Vogeli^52^. Each sample was reacted in the presence of 10 kPa BrF_5_, after which purified O_2_ gas was collected onto a 5 Å molecular sieve within a glass storage bottle. Carbonate separates were analysed using a conventional carbonate line, where purified CO_2_ gas was extracted after reacting the samples with 100% phosphoric acid^53^. Oxygen isotope ratios of magnetite, quartz and carbonates, and carbon isotope ratios of carbonates, were measured off-line using a Finnigan Delta XP mass spectrometer in dual-inlet mode. For magnetite and quartz analysed using laser fluorination, the Monastery Garnet standard^54^ was used to normalise the raw data and to correct for drift. Carbonates were analysed alongside the Namaqualand Marble standard^55^, and the raw data was corrected based on the calcite:dolomite ratio determined previously for each sample by XRD. The δ^18^O data are reported in per mil (‰) relative to the Standard Mean Ocean Water (SMOW) standard, and the δ^13^C data in per mil relative to the Pee Dee Belemnite (PDB) standard. Both types of analyses gave 2σ errors of 0.2‰.

## Data Availability

The authors declare that all relevant data are available within the article.
